# Supplementary material for: Single molecule mechanics resolves the earliest events in force generation by cardiac myosin
Source: eLife. 2019 Sep 17;8:e49266. doi: 10.7554/eLife.49266 (PMC6748826; doi:10.7554/eLife.49266)
Supplement: Supplementary file 1. [file elife-49266-supp1.docx]

**Supplementary Information for:**

**Single molecule mechanics resolves the earliest events in force generation by cardiac myosin**

Michael S. Woody^1^, Donald A. Winkelmann^2^, Marco Capitanio^3,4^, E. Michael Ostap^5*^_,_ Yale E. Goldman^5*^

^1^Graduate Group in Biochemistry and Molecular Biophysics, Perelman School of Medicine, University of Pennsylvania, Philadelphia, PA, USA, ^2^Department of Pathology and Laboratory Medicine, Robert Wood Johnson Medical School, Rutgers University, Piscataway, NJ, USA. ^3^LENS - European Laboratory for Non-linear Spectroscopy, Via Nello Carrara 1, 50019 Sesto Fiorentino, Italy. ^4^Department of Physics and Astronomy, University of Florence, Via Sansone 1, 50019 Sesto Fiorentino, Italy. ^5^Pennsylvania Muscle Institute, Perelman School of Medicine, University of Pennsylvania, Philadelphia, PA, USA

*Co-Corresponding authors to whom correspondence should be addressed

# **Supplementary Tables**

| **1 μM MgATP** | | | | | | | |
| --- | --- | --- | --- | --- | --- | --- | --- |
| **P_i_ (mM)** | **Force (pN)** | **N molecules** | **n events assisting** | **n events hindering** | **n events hindering <5 ms** | **n events hindering >15 ms** | **n events hindering >25 ms** |
| 0 | 0 | 20 | 1347 | |  |  |  |
|  | 1.5 | 6 | 790 | 952 | 72 | 819 | 778 |
|  | 2.25 | 5 | 963 | 1261 | 152 | 986 | 915 |
|  | 3 | 7 | 947 | 1340 | 457 | 506 | 471 |
|  | 3.75 | 5 | 1007 | 1502 | 572 | 906 | 871 |
|  | 4.5 | 6 | 970 | 1407 | 728 | 660 | 625 |
| 10 | 0 | 15 | 2225 | |  |  |  |
|  | 1.5 | 8 | 893 | 1114 | 115 | 928 | 893 |
|  | 2.25 | 5 | 1019 | 1049 | 228 | 769 | 730 |
|  | 3 | 5 | 843 | 991 | 338 | 583 | 552 |
|  | 3.75 | 6 | 784 | 1056 | 443 | 605 | 589 |
|  | 4.5 | 6 | 1047 | 1374 | 641 | 693 | 684 |
|  |  |  |  |  |  |  |  |
| **1 mM MgATP** | | | | |  |  |  |
| **P_i_ (mM)** | **Force (pN)** | **N molecules** | **n events assisting** | **n events hindering** |  |  |  |
| 0 | 0 | 7 | 267 | |  |  |  |
|  | 1.5 | 4 | 256 | 247 |  |  |  |
|  | 2.25 | 2 | 480 | 495 |  |  |  |
|  | 3 | 3 | 347 | 311 |  |  |  |

## Supplementary Table 1. Numbers of molecules, N, and actomyosin events, n, for each experimental condition.

| **0 P_i_** | | **10 mM P_i_** | |
| --- | --- | --- | --- |
| **Stroke Rate (s^-1^)** | ***k*_fast_ (s^-1^)** | **Stroke Rate (s^-1^)** | ***k*_fast_ (s^-1^)** |
| 837 | 676 | 1046 | 771 |
| 3176 | 1571 | 3074 | 986 |
| 5840 | 1593 | 4565 | 3273 |
| 4163 | 2430 | 4090 | 2025 |
| 4560 | 2696 | 5575 | 4044 |

## Supplementary Table 2. Comparison of observed stroke rates from Fig. 4c and observed fast detachment rates (*k*_fast_) from Fig. 2f.

|  | KCl | MgCl_2_ | CaCl_2_ | KH_2_PO_4_ | KOH | ATP | Na^+1^ (from ATP stock) | Ionic Strength |
| --- | --- | --- | --- | --- | --- | --- | --- | --- |
| 0 mM P_i_, 1 µM ATP | 25 | 1 | 1 | 0 | 0 | 0.001 | 0.002 | 31 |
| 10 mM P_i_, 1 µM ATP | 0 | 2* | 1 | 10 | 17 | 0.001 | 0.002 | 32.1 |
| 0 mM P_i_, 1 mM ATP | 16 | 2 | 1 | 0 | 0 | 1 | 2 | 31.2 |

All numbers are given in mM

## Supplementary Table 3. Solution conditions for experiments. All solutions contained 60 mM MOPS, 10 mM DTT, 1 mM EGTA, and 1 mg/mL BSA and an oxygen scavenging system in addition to what is listed in the table above.

*An extra 1 mM MgCl_2_ was added to the 10 mM P_i_ buffer to maintain approximately 1 mM free MgCl_2 ­_due to the coordination between P_i_ and MgCl_2._
